# Construction and applications of exosome-microneedle integrated systems

**DOI:** 10.1016/j.ijpx.2025.100360

**Published:** 2025-07-15

**Authors:** Lijie Zheng, Jiting Sun, Lusheng Wang, Zhixian Ding, Yu Tang, Mike Dai, Heng Tang

**Affiliations:** a Central Laboratory, Wanbei Coal Electric Group General Hospital, Suzhou 234000, China; b Laboratory of Inflammation and Repair of Liver Injury and Tumor Immunity, Hefei 230000, China

**Keywords:** Exosomes, Microneedles, Drug delivery, Disease therapy, Transdermal administration

## Abstract

With the rapid advancement of drug delivery technologies, microneedles (MNs) have emerged as a novel transdermal delivery platform due to their ease of administration, minimally invasive nature, and high efficiency. MNs have demonstrated broad applicability for delivering diverse therapeutic agents, including small molecules, nucleic acids, peptides, and proteins. Exosomes (Exos), a class of extracellular vesicles with unique biological functions and significant clinical potential, have attracted increasing attention in recent years. However, their widespread application is limited by issues such as poor stability, low delivery efficiency, and potential safety and immune risks. The integration of Exos with MNs (Exos-MNs) systems offers a promising strategy to address these challenges. This review provides a comprehensive overview of recent advances in Exos-MNs delivery systems, including the technological advances of MNs, biological characteristics and engineering strategy of Exos, and the construction strategies of Exos-MNs. Additionally, we highlight recent developments in the application of Exos-MNs systems and discuss future perspectives and challenges for their clinical translation.

## Highlights

• Exos-MNs systems integrate Exos bioactivity with MNs delivery efficiency.
• Exos-MNs address Exos instability and limited delivery efficiency challenges.
• Exos-MNs show therapeutic potential in wound healing, organ ischemia/reperfusion injuries, and canker sores.
• Technological advances in Exos-MNs design and construction are comprehensively reviewed.
• Clinical translation challenges, including safety and standardization, are critically discussed.

## 1 Introduction

Exosomes (Exos) are extracellular vesicles with diameters ranging from 30 to 150 nm, secreted by various cell types and characterized by a lipid bilayer membrane structure. These vesicles play crucial roles in intercellular communication and serve as promising drug delivery carriers. They are widely distributed in diverse bodily fluids including blood, saliva, and urine (Goodman, 2025). Exos carry various bioactive molecules such as proteins, mRNAs, miRNAs, and lipids, demonstrating therapeutic potential for multiple diseases including Alzheimer's disease, myocardial infarction, retinal disorders, skin wound healing, ischemic stroke, and pancreatic diseases (Tajabadi et al., 2025).

**Fig. 1:**
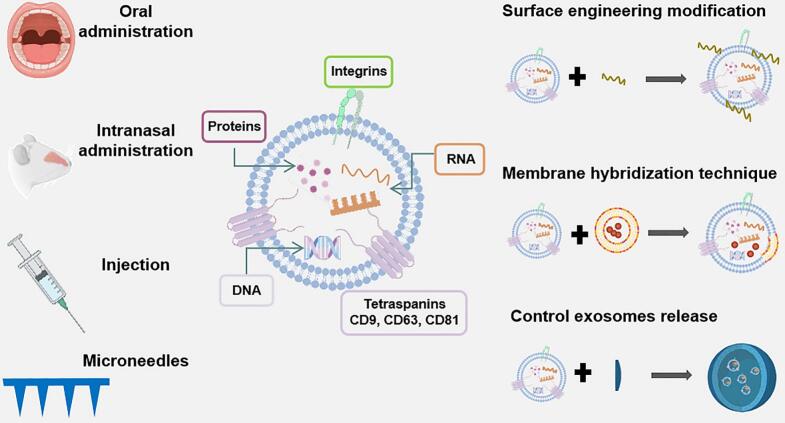
Structure of Exos, optimization strategies and schematic diagram of delivery routes for Exos. The Exos surface engineering modification, membrane hybridization technology and pharmaceutics are used to prolong the half-life and enhance the stability in vivo, and Exos are usually delivered by oral administration, intranasal administration, injection and MNs.

Short half-life and in vivo instability are the main reasons affecting the effective enrichment and therapeutic efficacy of Exos. The modification of Exos surfaces (Lin et al., 2025)、enhancement of Exos structure, slow-release design and delivery system optimisation are beneficial to prolong in vivo half-life and improve stability, as shown in Fig. 1. Surface modification of Exos effectively improves the surface properties of Exos through physical, chemical and biological methods to achieve better performance in drug delivery systems. For example, the introduction of polyethylene glycol (PEG) molecules into the Exos surface, and the formation of PEGylated Exos by covalent bonding can significantly improve the stability and biocompatibility of Exos and prolong their circulation time in vivo (Fukuta et al., 2023). Strengthening the Exos structure by membrane hybridisation techniques, such as the synthesis of liposome-fused Exos, increases membrane stiffness, resists shear forces and increases the drug delivery capacity of Exos (Zhou et al., 2024). The principles of formulation science for sustained release design provide longer lasting effects for Exos drug delivery systems. Environmentally sensitive delivery systems are designed based on the physiological environment in the body. For example, pH-sensitive sustained release formulations are being developed for rapid drug release in the acidic environment of tumor tissues and slow release in normal tissues (Yuan et al., 2025). However, there are significant limitations to the effective delivery of Exos. Although several administration routes, such as oral, injection, and intranasal, have been successfully employed, these methods may not allow Exos to stay at the lesion site long enough to achieve satisfactory effects. Optimisation strategies for Exos delivery systems further enhance their value in drug delivery where local administration replaces systemic administration to reduce systemic toxicity and side effects, e.g. Exos embedded in microneedles (MNs) for localized slow release in lesions (Chen et al., 2024a).

MNs technology, an emerging method of transdermal drug delivery, consists of multiple arrays of tiny needles, ranging in needle length from a few hundred microns to a few millimetres, attached to a base. It has already shown potential for a wide range of applications in the treatment of a variety of conditions such as superficial skin tumors, proliferative scarring, arthritis, and the delivery of vaccines and insulin. MNs are applied to the skin in a minimally invasive manner to draw up interstitial fluids in the skin or to facilitate the transdermal delivery of drugs containing components such as small molecules, nucleic acids, peptides and therapeutic cells, and they also avoid contact with nerve endings in the skin to reduce pain sensation (Balde et al., 2025). Recent studies have shown that Exos can be delivered efficiently using MNs technology, enhancing local therapeutic effects and improving patient compliance (Nguyen, 2025; Xu et al., 2024). Therefore, the drug delivery system based on MNs technology can penetrate the skin barrier to achieve efficient drug delivery, which is expected to solve the challenges of pain, potential damage, and non-durable administration of Exos due to the delivery method, and provide a promising strategy for the delivery of Exos.

Although the integration of Exos with MNs (Exos-MNs) has been previously summarized in recent reviews (Wang et al., 2024e; Fathi-Karkan et al., 2023), those articles primarily focus on the fabrication of MNs, Exos synthesis and characterization, as well as the local and sustained delivery capabilities of Exos-MNs systems. However, key technical challenges—including Exos stability, biosafety, and precise spatiotemporal release within MNs systems—have been only briefly addressed. In this review, we provide an updated and focused overview of recent advances in Exos-MNs platforms, with particular emphasis on system design, fabrication strategies, and therapeutic applications across various disease models. Furthermore, we discuss current limitations and future perspectives, aiming to inspire innovative research and offer a valuable reference for researchers working in this emerging field.

## 2 Progress in the study of drug delivery systems for MNs

The development of MNs technology as an innovation in transdermal drug delivery marks the evolution from a single solid MNs (SMNs) to multiple MNs systems. The advancement of this technology reflects not only the development of materials science and micro-nano processing technology, but also the continuous pursuit of drug delivery efficiency and patient compliance.

### 2.1 History of MNs technology

The development of MNs technology can be traced back to the middle of the 20th century, and was initially proposed as part of the Transdermal Drug Delivery System. In 1958, Alan Richard Wagner, USA, introduced the concept of intradermal injection of MNs. It was not until 1998 that Henry et al., Georgia Institute of Technology, USA, first used MNs for transdermal drug delivery studies. This study formally introduced MNs technology into the field of drug delivery and triggered a boom in the development of MNs (Henry et al., 1998). By 2020, MNs had been selected as one of the top 10 emerging technologies by Scientific American and the World Economic Forum.

Development of MNs technology has undergone an evolution from SMNs to multiple types of MNs (Sabbagh and Kim, 2022). Initially, SMNs were usually made of metals (e.g., stainless steel or titanium) and non-degradable polymers, etc. And were mainly used as skin pre-treatment tools for transdermal drug delivery to improve the efficiency of transdermal drug delivery by piercing the skin to form microporous channels. With the deepening of research, the technology of MNs began to diversify, including Coated MNs (CMNs), Hollow MNs (HMNs), Dissolving MNs (DMNs) and Hydrogel-Forming MNs (HFMNs), etc. The emergence of these novel MNs not only improves the efficiency and accuracy of drug delivery, but also expands the application scope of MNs technology.

Today, MNs are increasingly being investigated in both preclinical and clinical settings, particularly for applications in vaccination, cancer therapy, wound healing, and chronic disease management. Their integration with advanced drug carriers such as nanoparticles, hydrogels, and Exos represents a new frontier in targeted and personalized medicine.

### 2.2 Types and characteristics of MNs

**Fig. 2:**
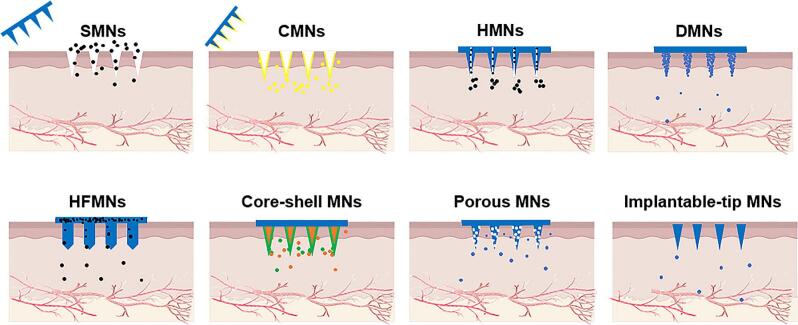
Different types of MNs and principles of drug release.

MNs can be broadly categorized based on structure and function: SMNs, CMNs, HMNs, DMNs, HFMNs, porous MNs, and implantable-tip MNs. An overview of these MNs types and their structures is presented in Fig. 2.

CMNs are developed on the basis of SMNs, which are coated with drugs or materials. When CMNs penetrate the skin, the drug enters the skin with the CMNs and then diffuses into the bloodstream for local or systemic drug delivery (Ingrole and Gill, 2019). HMNs are similar to micron-sized micro-syringes. These MNs can pre-load drugs into the hollow structure of the needle body, and after piercing the skin through the HMNs, they enter the body driven by the pressure of the concentration gradient of the tissue fluid to achieve drug delivery (Nadda and Das, 2025). It has the dual characteristics of injection drug delivery and transdermal drug delivery. DMNs are made from a mixture of biodissolvable materials and drugs. After penetrating the stratum corneum of the skin, the drug components are released as the DMNs dissolve, allowing the drug to penetrate and be absorbed into the subcutaneous tissue and the human body (Wang et al., 2023).

HFMNs composed of crosslinked hydrophilic polymers have attracted growing interest in the field of transdermal drug delivery. Unlike other MNs types that encapsulate drugs within the needles themselves, HFMNs function through a distinct mechanism: upon insertion into the skin, the HFMNs rapidly absorb interstitial fluid and swell, forming continuous aqueous channels that connect the dermal microenvironment with a drug-loaded backing layer (Turner et al., 2021). Through these hydrogel conduits, therapeutic agents contained in the external reservoir can diffuse into the surrounding tissue in a controlled manner. Notably, in addition to enabling sustained drug delivery, HFMNs are also capable of extracting interstitial skin fluid, thereby offering dual functionality for both therapeutic and diagnostic applications.

In addition, core -shell MNs represent another specialized design, featuring a layered structure that enables sequential drug release, making them suitable for complex delivery regimens (Zhang et al., 2023).Porous MNs fabricated with interconnected micropores or nanochannels, support bidirectional transport across the skin, enabling both real-time fluid sampling and localized drug delivery (Bao et al., 2022; Gao et al., 2023). Implantable-tip MNs are designed to embed drug-loaded tips within the dermis, allowing for prolonged retention and sustained release (Lee et al., 2021; Li et al., 2025). These systems are particularly advantageous for long-acting therapies and can be further engineered for stimuli-responsive release triggered by environmental cues such as pH, temperature, or enzymes.

The design of bionic MNs is inspired by nature, mimicking the structure and function of living organisms to improve the performance of MNs. Notably, these biomimetic strategies are typically implemented using existing MNs formats, such as DMNs, or HFMNs, rather than representing a distinct structural category. For instance, mosquito-inspired MNs mimic the insect's painless blood-sucking mechanism, leading to the development of ultra-fine needles (∼60 μm in diameter) with excellent penetration and minimal pain (Zhou et al., 2023). Similarly, bee-stinger-inspired MNs feature barbed structures that improve skin insertion and enhance tissue adhesion, offering new strategies for stable transdermal drug delivery (Zhang et al., 2022a, Zhang et al., 2022b). These bioinspired designs, although categorized under conventional MNs types, provide innovative strategies to optimize delivery efficiency, patient comfort, and device-tissue interaction.

### 2.3 Application of novel materials in the preparation of MNs

The development of novel materials has had a significant impact on the performance of MNs technology, improving not only the biocompatibility and biodegradability of MNs, but also the efficiency and accuracy of drug delivery. The properties of these materials allow MNs to be better adapted to specific therapeutic environments and needs, opening up new opportunities in areas such as drug delivery, vaccination and bioassays.

The use of biocompatible materials in MNs technology plays a crucial role in enhancing safety and environmental sustainability. Notably, DMNs are typically fabricated from fast-dissolving hydrophilic polymers such as hyaluronic acid (HA) and polyvinylpyrrolidone (PVP), which rapidly dissolve upon skin insertion to release therapeutic agents. For example, dual-layer DMNs prepared from HA with excellent biosafety and dual antimicrobial and antioxidant activity to meet the diverse needs of the diabetic wound healing process (Liu et al., 2023). In contrast, biodegradable MNs are constructed from slowly degrading polymers such poly (lactic-*co*-glycolic acid) (PLGA), which remain in the tissue for extended periods and release drugs in a sustained manner. These materials degrade through hydrolysis over days to weeks, making them suitable for long-acting or controlled-release applications. Furthermore, the incorporation of stimuli-responsive materials, such as temperature-sensitive or pH-sensitive polymers, has enabled the design of MNs capable of releasing drugs in response to specific physiological or pathological cues. For instance, pH-sensitive MNs have been developed to achieve rapid drug release in the acidic microenvironment of tumors, offering a promising strategy for targeted cancer therapy (Makvandi et al., 2021; Tang et al., 2024).

Materials with good electrical conductivity and biocompatibility can be used to prepare iontophoresis MNs, a new type of MNs that utilizes an electric field to drive the drug through the skin, improving drug penetration and delivery efficiency (Wang et al., 2024a). In addition, the use of nanomaterials such as graphene improves the sensitivity and responsiveness of the sensors, and the preparation of MNs biosensors using these materials enables minimally invasive sampling of subcutaneous interstitial fluid for continuous and real-time monitoring (Wang et al., 2024b).

## 3 Exos biology and engineering strategies

### 3.1 Types and sources of Exos

**Fig. 3:**
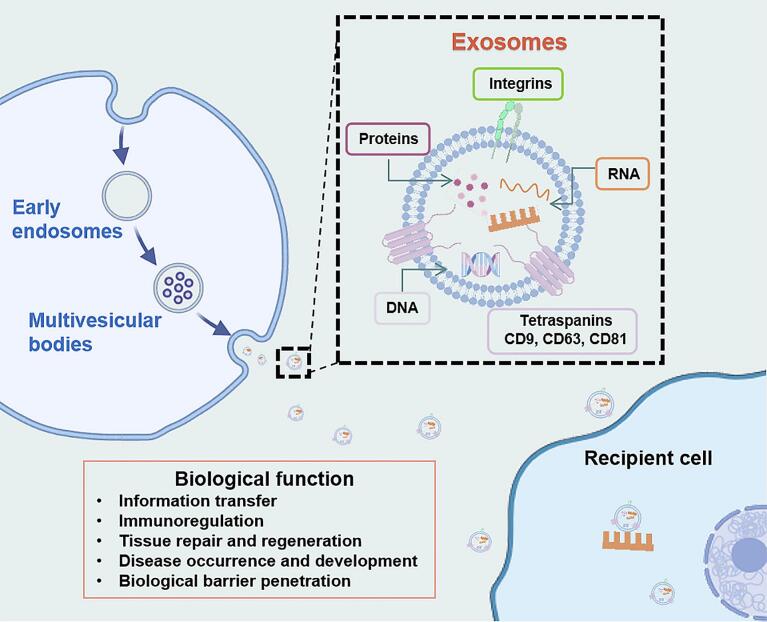
Physiological process, structural characteristics and physiological functions of Exos.

As illustrated in Fig. 3, Exos are nanosized extracellular vesicles originating from the inward budding of the endosomal membrane, leading to the formation of multivesicular bodies (MVBs). Upon fusion of MVBs with the plasma membrane, Exos are released into the extracellular space. Structurally, Exos possess a lipid bilayer enriched with cholesterol, sphingomyelin, and specific membrane proteins such as tetraspanins (CD9, CD63, CD81) and integrins. Beyond their vesicular architecture, the biological activity of Exos primarily stems from the diverse cargo they encapsulate, including proteins, DNA, mRNA, miRNA, and lipids. These molecular components play a pivotal role in mediating intercellular communication and regulating a wide range of physiological and pathological processes, such as immune modulation, tissue repair, tumor progression, and the ability to cross biological barriers.

Exos are secreted by virtually all cell types, but their biological properties and therapeutic potential vary significantly depending on their cellular origin. Here, we compare Exos from common sources, including those derived from mesenchymal stem cells (MSCs), immune cells, and tumor cells. MSC-derived Exos (MSC-Exos) are widely studied for their regenerative and immunomodulatory effects. They carry anti-inflammatory cytokines, growth factors (e.g., TGF-β, VEGF), and miRNAs that promote tissue repair and immune regulation. Their low immunogenicity and high biocompatibility make them promising for clinical applications in wound healing, cardiovascular, and neurodegenerative diseases (Abdulmalek et al., 2024). Immune cell-derived Exos from dendritic cells, macrophages, and T cells play key roles in immune modulation. For example, DC-derived Exos present antigens to T cells, while macrophage-derived Exos may exert pro- or anti-inflammatory effects depending on polarization (Morisaki et al., 2025; Gharavi et al., 2022). Tumor-derived Exos (TEXs) contain oncogenic proteins, miRNAs (e.g., miR-21, miR-210), and immunosuppressive molecules. They facilitate tumor progression by promoting angiogenesis, immune evasion, and metastasis. Despite their pathological role, TEXs are also explored as biomarkers and engineered delivery vehicles for targeted therapy (Yu et al., 2025).

### 3.2 Isolation and purification techniques

The quality and consistency of Exos are largely influenced by the isolation method employed. Commonly used techniques include differential ultracentrifugation, size-exclusion chromatography (SEC), tangential flow filtration (TFF), immunoaffinity-based capture, and polymer precipitation (Guan et al., 2020; Chen et al., 2021).

Among these, differential ultracentrifugation is the most widely used and is often considered the gold standard for Exos isolation (Coughlan et al., 2020). It yields relatively high-purity Exos but is time-consuming and has limited scalability. SEC preserves vesicle integrity and ensures uniform size distribution without significantly altering biological properties. However, it may co-isolate similarly sized contaminants, reducing overall purity. TFF is a scalable and GMP-compliant approach that is increasingly adopted for clinical-grade Exos production. Nonetheless, it requires specialized equipment and trained personnel. Polymer precipitation offers a simple and rapid procedure, but generally results in lower purity due to co-precipitation of contaminants (Kamei et al., 2021). Immunoaffinity-based capture enables the selective isolation of specific Exos subpopulations but may alter surface characteristics, potentially affecting downstream applications. Each isolation strategy requires careful consideration of trade-offs between purity, yield, scalability, and compatibility with downstream applications such as drug loading or MNs fabrication.

### 3.3 Drug loading techniques

To enhance the therapeutic efficacy of Exos, various drug loading strategies have been developed to incorporate small molecules, nucleic acids, proteins, or nanoparticles into Exos (Luan et al., 2017; Vader et al., 2016). These methods can be broadly classified into endogenous and exogenous loading techniques, depending on whether the cargo is introduced before or after Exos isolation (Gul et al., 2024; Zeng et al., 2023b). Table 1 provides an overview of the major drug loading strategies for Exos, highlighting their advantages, limitations, and suitable cargo types.

**Table 1:** Comparison of Endogenous and Exogenous Exos Loading Techniques.

Loading Strategy	Method	Advantages	Limitations	Suitable Cargo Types
Endogenous	Transfection	Stable expression of therapeutic RNA/protein; high specificity	Low yield; time-consuming; may affect cell viability	miRNA, mRNA, proteins
	Drug incubation	Simple; maintains Exos integrity	Limited loading efficiency; passive uptake only	Small-molecule drugs (lipophilic)
Exogenous	Passive incubation	Simple; non-disruptive	Low loading efficiency; only suitable for lipophilic cargo	Small lipophilic drugs
	Electroporation	Enables RNA/protein loading	Risk of aggregation; vesicle membrane disruption	siRNA, miRNA, mRNA, proteins
	Sonication	High loading efficiency; suitable for hydrophilic drugs	May damage vesicle structure and bioactivity	Hydrophilic drugs, some proteins
	Freeze–thaw cycles	Gentle method; improves membrane permeability	Risk of cargo or membrane denaturation	Small molecules, proteins
	Extrusion	Promotes physical mixing; uniform vesicle size	Alters morphology; requires specialized equipment	Nanoparticles, proteins
	Chemical transfection	Efficient for nucleic acid delivery	Potential cytotoxicity from reagents (e.g., lipofectamine)	RNA, DNA, gene-editing tools (e.g., CRISPR)

Endogenous loading involves engineering donor cells to produce Exos that inherently carry the desired therapeutic cargo. This can be achieved by transfecting of donor cells with plasmids encoding therapeutic RNAs or proteins, or by incubating the cells with small-molecule drugs that are passively incorporated into Exos during biogenesis (Liu et al., 2019; Biton et al., 2019). This method ensures efficient encapsulation and preserves Exos integrity but may have limitations in loading capacity and scalability (Lamichhane et al., 2015).

Exogenous loading involves the introduction of therapeutic agents into isolated Exos using physical or chemical methods (Mehryab et al., 2020b). For example, passive incubation is a simple and non-disruptive technique suitable for lipophilic small molecules. Electroporation applies electrical pulses to transiently open the Exos membrane, enabling the loading of RNAs or proteins, although it may lead to aggregation or structural disruption (Liang et al., 2020; Kooijmans et al., 2013). Sonication temporarily destabilizes the membrane using ultrasound, enhancing loading efficiency—particularly for hydrophilic drugs (Gao et al., 2021). Freeze-thaw cycles repeatedly freeze and thaw Exos to permeabilize the membrane and facilitate cargo encapsulation; this method is relatively gentle but may denature sensitive cargo or vesicle components. Saponin permeabilization or chemical conjugation: Used to enhance membrane permeability or covalently link drugs to the Exos surface. Extrusion and transfection are more advanced strategies (Zhang et al., 2022b). Extrusion forces Exos-cargo mixtures through membranes with defined pore sizes, promoting physical mixing, while chemical transfection methods (e.g., using lipofectamine) enable intracellular cargo delivery but introduce potential toxicity concerns (Mehryab et al., 2020a, Mehryab et al., 2020b).

Each technique presents trade-offs in terms of loading efficiency, cargo stability, vesicle integrity, and clinical translatability. The optimal loading strategy should be selected based on the physicochemical properties of the therapeutic cargo and the intended application.

### 3.4 Exos characterization techniques

Exos characterization is essential for understanding their biophysical properties, cargo composition, and potential applications in diagnostics and therapeutics (Fang et al., 2022). A comprehensive evaluation typically involves multiple techniques to assess size, morphology, molecular content, and surface markers. The choice of method depends on the specific research objectives and available resources. Commonly used methods include nanoparticle tracking analysis (NTA), which measures size distribution and concentration; Transmission electron microscopy (TEM), which provides detailed visualization of Exos morphology and verifies structural integrity; and western blotting/flow cytometry to confirm the presence of characteristic surface markers (e.g. CD9, CD63 and CD81).

Each method has its own strengths and limitations, so multiple techniques are often used in combination to gain a comprehensive understanding of Exos properties.

## 4 Study of MNs technical delivery of Exos

Derived from autologous cells, Exos have low immunogenicity, high biocompatibility and stability in body fluids, making them promising therapeutic agents and drug carriers (Xu et al., 2019). However, their clinical translation faces several challenges, including poor in vitro stability during long-term storage, low in vivo delivery efficiency, limited target site retention. To address these challenges, various delivery enhancement strategies have been explored, including the use of MNs platforms and nanomaterial-based systems. MNs, in particular, have shown promise in improving Exos delivery by enabling minimally invasive, localized transdermal administration (Taghavi et al., 2025).However, it should be noted that the improved stability of Exos primarily arises from the protective polymer matrix used in MNs fabrication, rather than the MNs format itself. Similarly, MNs do not inherently enhance the targeting properties of Exos, except in specific scenarios such as lymphatic delivery or localized targeting of skin or tissue.

### 4.1 Strategies for the construction of Exos-MNs

The combination of Exos with MNs provides a new solution for the efficient delivery of Exos. By loading Exos into MNs, localized, targeted and controlled release of Exos can be achieved, thereby enhancing its therapeutic effect. Common strategies for the construction of Exos-MNs include encapsulation, physical adsorption, and chemical cross-linking.

The embedding method homogeneously mixes Exos directly with MNs material to form Exos-MNs system with high loading and uniform distribution. It is commonly used to prepare DMNs and HFMNs. In addition, the biological activity of Exos can be maintained and long-term storage can be achieved by making MNs by freeze-drying technique. For example, Exos can be mixed with materials such as HA or PVP and freeze dried to form DMNs (Wen et al., 2024).

The physical adsorption method is to adsorb Exos on the surface or inside of MNs by physical action (e.g. electrostatic adsorption, hydrophobic interactions, etc.). This method is simple to use and does not significantly affect the biological activity of Exos. It is commonly used for the preparation of CMNs, e.g. Wang et al. prepared viscous MNs for the treatment of uterine adhesions (Wang et al., 2024c).

The chemical cross-linking method combines Exos with MNs materials through chemical bonding (e.g. covalent bonding, hydrogen bonding, etc.). This method can increase the bond strength between Exos and MNs, resulting in more stable loading and controlled release. It is commonly used in the preparation of DMNs and HFMNs. For example, Exos has been covalently linked to aminated HA to form stable complexes (Wen et al., 2024).

The embedding method is easy to operate and preserves the natural structure of Exos, but the sudden release effect may affect its slow release; the physical adsorption method is easy to operate but the loading is limited; the chemical cross-linking method has a large loading but may affect the biological activity of Exos; and the freeze-drying method is suitable for long-term storage but complicated to operate. Future studies should combine the advantages of different strategies to develop a more efficient, stable and controllable delivery system for Exos-MNs to meet the needs of clinical therapy.

### 4.2 MNs delivery mechanism of Exos

Physical facilitation mechanism: MNs physically form micropores on the skin surface, which bypass the cuticle and provide a direct pathway for Exos to enter the skin (Li et al., 2023a). Exos passes through the micropores and enters directly into the epidermis or upper dermis, thus participating in microcirculation and exerting a pharmacological response, and this physical facilitation significantly improves the efficiency of transdermal delivery of Exos. In addition, different shapes of MNs can be designed to improve the delivery efficiency (Li et al., 2023b), such as cone-shaped MNs usually have a sharp tip, which can easily penetrate the surface layer of the skin and effectively improve the efficiency of transdermal drug delivery. Pyramid-shaped MNs have a pyramid-like structure of polyhedral MNs, and this structure can help improve the stability and drug loading capacity of MNs. The tips of arrowhead-shaped MNs were designed in the shape of an arrowhead, which could improve penetration and drug delivery efficiency.

Painless drug delivery mechanism: The use of MNs significantly reduces the tissue damage and pain associated with traditional injections due to the micron size of the MNs. The MNs do not trigger the nociceptors when piercing the skin, thus allowing for painless drug delivery, significantly reducing the tissue damage and pain associated with traditional injections. This technology combines the non-invasiveness of traditional transdermal drug delivery with the efficiency of injectable drug delivery, providing a completely new approach to drug delivery (Lin et al., 2022).

Double carrier delivery mechanism: In addition to its direct use as a therapeutic agent, Exos can also be used as a drug carrier, integrating with MNs to form a dual carrier system that protects the drug from in vivo metabolism and improves drug targeting. The integration of extracellular vesicles encapsulating mRNA into MNs for collagen replacement therapy has shown that intradermal delivery via MNs results in better distribution of mRNA in the dermis and subcutis and longer retention of collagen (You et al., 2023). Exos-MNs provide multiple delivery options for Exos, which can achieve precise drug release and efficient therapy (Hu et al., 2023). In the future, with further optimisation of MNs technology, transdermal delivery of Exos will play a greater role in disease treatment.

## 5 Advantages of MNs delivering Exos

### 5.1 Improving the stability of Exos

Exos are less stable in vitro, especially during long-term storage, and tend to lose their biological activity and efficacy, which severely limits their clinical applications (Yang et al., 2025). To address this problem, researchers have developed a variety of storage methods, including lyophilisation, cryopreservation and spray drying. However, it has been found that Exos are still susceptible to degradation or inactivation when stored as a liquid or lyophilised preparation in an ordinary environment. Therefore, the development of a storage method that can maintain the activity of Exos for a long period of time has become an urgent need for current research.

The MNs technology offers new ideas for solving the stability problem of Exos. It has been shown that polymer-based MNs are capable of preserving the activity of various sensitive bioactive substances (e.g. proteins, DNA and cells). For example, bee venom peptides remained stable for more than 1 month when loaded into HA-based MNs (Zheng et al., 2024). MNs influenza vaccine retains most of its activity after 6 months of storage at 4 °C and 25 °C (Vander et al., 2024). And cryo-MNs fabricated via sequential cryomolding successfully preserved and delivered live therapeutic cells (Chang et al., 2021). These studies suggest that the polymer-based MNs matrix can act as an effective stabilizing environment for Exos.

Given that Exos are biologically active vesicles with complex surface and cargo compositions, long-term preservation requires tailored material selection. For example, sugar-based MNs have been reported to maintain Exos activity while facilitating intradermal delivery for enhanced immunotherapeutic outcomes (Mu et al., 2024). In addition, the use of alginate as an excipient for MNs effectively prevents aggregation and cryo-damage of Exos, thus facilitating the long-term storage of Exos in MNs (Charoenviriyakul et al., 2018). In summary, the solid-state polymer formulation of MNs offers a practical solution for storage, potentially reducing reliance on cold-chain logistics and expanding the feasibility of Exos-based therapies.

### 5.2 Enhancing the security of Exos

After insertion into the skin, MNs form transient and reversible microchannels in the stratum corneum. Although this process may cause mild erythema or allergic reactions, such adverse effects can be significantly minimized by using biocompatible materials for MNs fabrication (Zhao et al., 2023a, Zhao et al., 2023b). Compared to conventional hypodermic needles, MNs pose lower risks and, due to their microscale dimensions, can penetrate the skin barrier without stimulating nociceptive nerves, thereby enabling pain-free drug delivery. This painless administration not only enhances patient comfort but also significantly improves treatment compliance.

As a natural nanoscale drug carrier, Exos has multiple physiological functions and good biocompatibility. Compared with conventional drugs, Exos can effectively avoid toxicity and immunogenicity problems, thus reducing the incidence of adverse reactions. When integrated into MNs systems, Exos can be locally administered to targeted sites, minimizing systemic distribution and further decreasing off-target toxicity.

### 5.3 Enhance the delivery efficiency of Exos

The clinical application of Exos is significantly limited by their inefficient accumulation at target site (Rezaie et al., 2022). Conventional delivery methods rely primarily on passive diffusion, resulting in suboptimal localization and reduced therapeutic efficacy. In the context of transdermal delivery, the stratum corneum poses a major barrier, limiting the penetration of topically applied Exos formulations and leading to low bioavailability. MNs technology offers a promising strategy to enhance the localized delivery of Exos.

MNs are capable of delivering Exos directly to the epidermis or dermis in the vicinity of the focal area by penetrating the stratum corneum of the skin. In addition, the length, size and shape of the MNs can be customised according to therapeutic needs to ensure effective delivery of Exos to the target site, significantly increasing the concentration of Exos in local tissues. This localized delivery not only reduces the distribution of Exos throughout the body, but also avoids their rapid clearance in the circulation, thereby enhancing their accumulation at the target site. However, it should be noted that MNs do not inherently enhance biological targeting properties of Exos. Improvements in targeting require additional strategies, such as surface modification of Exos with ligands or antibodies that facilitate recognition and uptake by specific cells or tissues (Li et al., 2024). In this context, MNs primarily serve as a minimally invasive delivery vehicle, enabling efficient dermal deposition, particularly for labile or poorly absorbed drugs.

### 5.4 Enabling controlled release of Exos

As an efficient drug delivery vehicle, the controllable release of Exos is critical for maximizing therapeutic efficacy. Traditional Exos delivery methods often lack precise release control, leading to rapid or uneven drug distribution in the body, which compromises treatment outcomes. The introduction of MNs technology provides a new solution for the controlled and sustained release of Exos. Through rational design of MNs materials and structures, various release profiles—including sustained, controlled, and stimuli-responsive release—can be achieved to meet disease-specific therapeutic needs (Yang et al., 2022).

MNs fabricated with temperature-, pH-, or enzyme-responsive materials enables the environmental-responsive release of Exos. For example, temperature-sensitive lipid gels can function as in situ drug reservoirs for encapsulating Exos, with drug release modulated by temperature and light (Zhao et al., 2023b). In particular, the use of temperature-sensitive MNs accelerated Exos release at elevated body temperature, a process that can be explored by engineering the thermal responsiveness of the MNs materials. Additionally, selecting materials with different degradation rates or designing core-shell structured MNs allows for differential and prolonged Exos release. This enables modulation of the degradation of the MNs, thereby fine-tuning the drug release rate (Lyu et al., 2024).The controlled release characteristics of Exos-MNs systems have shown substantial therapeutic advantages across a range of disease models. For instance, MNs fabricated using silk fibroin methacryloyl can achieve sustained Exos release, effectively promoting wound healing and tissue regeneration (Hu et al., 2024). Furthermore, layered MNs composed of gelatin methacrylate and sodium alginate enable sequential release of Exos and co-delivered therapeutics, offering improved control over disease progression (Chen et al., 2024b). To better understand how the selection of MNs materials influences therapeutic outcomes, Table 2 summarizes commonly used polymers and stimuli-responsive materials, their respective drug release mechanisms, and representative biomedical applications.

**Table 2:** Summary of commonly used MNs matrix materials, their release behavior, stimuli-responsiveness, and applicable disease types.

Material type	Release behavior	Responsive Behavior	Representative application	Reference
HA	Rapid dissolution and release	No	Skin rejuvenation, superficial drug delivery	(Zhao et al., 2018)
PLGA	Time-dependent biodegradation	No	Long-term delivery for chronic disease treatment	(Chen et al., 2023)
Silk fibroin methacryloyl	Sustained release via slow material degradation	No	intervertebral disc degeneration and tissue regeneration	(Hu et al., 2024)
GelMA + alginate	Sequential/stage-wise release	Swelling upon contact with fluid	Multimodal therapy for chronic wounds	(Chen et al., 2024b)
Temperature-sensitive materials	Release controlled	Thermo-responsive (e.g., 1ight or temperature)	Repair of bone defects	(Zhao et al., 2023b)
pH-sensitive materials	Acid-triggered release	pH- responsive	Targeted therapy for tumor or inflammatory lesions	(Huang et al., 2022)
Enzyme-responsive polymer	Enzyme-triggered degradation and drug release	enzyme-responsive	Infection-triggered immunomodulation	(Yang et al., 2024)

## 6 Application of Exos-MNs

**Fig. 4:**
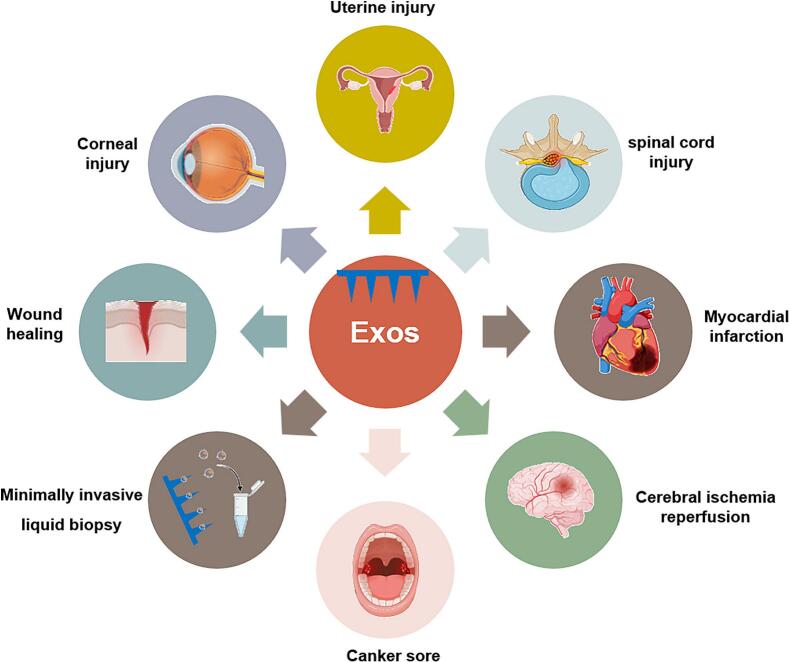
Schematic illustration of the biomedical applications of Exos-MNs systems. The selected applications include: Minimally invasive liquid biopsy, Wound healing and tissue regeneration (e.g., skin, corneal, uterine, and spinal cord injuries), Organ ischemia-reperfusion injuries (e.g., myocardial and cerebral), and canker sore treatment.

Although still in the preclinical stage, the Exos-MNs system demonstrates significant therapeutic potential across a broad spectrum of biomedical applications. As illustrated in Fig. 4, Exos-MNs have been explored in various fields, including minimally invasive liquid biopsy; wound healing and tissue regeneration (such as skin, corneal, uterine, and spinal cord injuries); organ ischemia/reperfusion injuries (e.g., cardiac and cerebral); and the treatment of canker sores.

To provide a comprehensive overview, the following sections will examine each application domain in detail, highlighting key delivery strategies, underlying therapeutic mechanisms, and observed outcomes.

### 6.1 Minimally invasive liquid biopsy

Liquid biopsy enables real-time, non-invasive molecular diagnostics and has become a cornerstone in precision medicine. Exos, as carriers of disease-specific molecular signatures, are widely recognized as potential biomarkers (Zeng et al., 2025). However, conventional Exos isolation methods are often invasive and time-consuming. To address this, Park et al. developed HFMNs for direct extraction of interstitial fluid (ISF), enabling early tumor diagnosis (Park et al., 2023). Upon insertion, the HFMNs swell and capture glypican-1-positive tumor-derived Exos via antibody interaction, facilitating efficient and minimally invasive biomarker collection.

### 6.2 Wound repair and tissue regeneration

Tissue injury is a common clinical problem that can cause a great burden to patients' lives. Exos are rich in a variety of bioactive substances, and with the release of these bioactive substances, Exos can reduce inflammation, promote angiogenesis, and accelerate cell proliferation and migration, which has shown great advantages in wound and tissue regeneration (Joorabloo and Liu, 2023). Due to their unique composition and structure, MNs-delivered Exos have attracted widespread attention in various tissue regeneration, including skin wounds, corneal injurie, uterine injurie and spinal cord injurie. The use of MNs for in situ delivery of bioactive molecules, MSCs and growth factors allows for targeted organisation and better spatial distribution. At the same time, MNs can provide mechanical support or targeted traction to the tissue, thereby accelerating tissue repair.

#### 6.2.1 Skin wounds

**Fig. 5:**
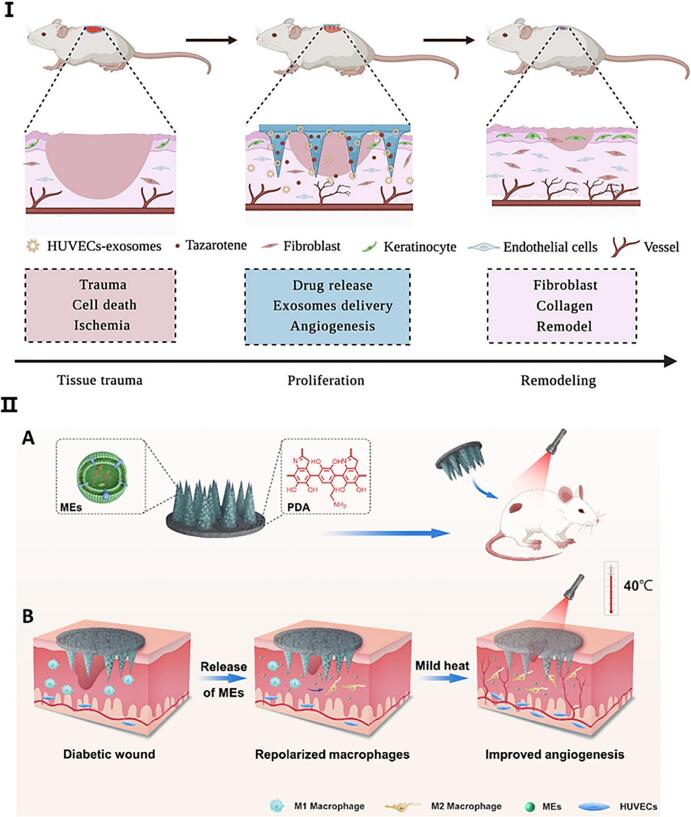
Exos-MNs for diabetic wound treatment. (I) GelMA/PEGDA MNs co-loaded with HUVECs-derived Exos and tazarotene enhances angiogenesis and accelerates wound healing. Adapted from (Yuan et al., 2022). (II) Schematic illustration of bilayer MNs (MEs-PMNs) delivering M2 macrophage-derived Exos and photosensitizer for synergistic immunomodulation and photothermal therapy in diabetic wounds. Adapted from (Zeng et al., 2023a, Zeng et al., 2023b).

Exos exhibit functional diversity depending on their cellular origin, reflecting the biological properties of their parent cells. To preserve their natural bioactivity and enhance therapeutic efficacy, various MNs platforms have been developed for precise and efficient delivery. One strategy involves co-loading human umbilical vein endothelial cell (HUVEC)-derived Exos and the wound healing agent tazarotene into methacrylate gelatin (GelMA)/PEG-based MNs for transdermal administration (Fig. 5I)(Yuan et al., 2022). This dual-loaded system facilitates targeted delivery of both Exos and drugs to the wound site, thereby promoting cell proliferation, migration, and angiogenesis in both in vitro and in vivo diabetic wound models.

To further enhance wound repair, Zeng et al. designed bilayer MNs capable of sequentially releasing Exos and a photosensitizer (Fig. 5II) (Zeng et al., 2023a, Zeng et al., 2023b). In this system, M2 macrophage-derived Exos are encapsulated in the needle tips and released directly into the wound to modulate the inflammatory microenvironment and stimulate angiogenesis. Meanwhile, the backing layer contains polydopamine (PDA) and a photosensitizer, which upon mild near-infrared irradiation generates localized heat, further promoting angiogenesis and granulation tissue formation. This synergistic approach significantly accelerates diabetic wound healing. Additionally, platelet-derived Exos—known for their anti-inflammatory and pro-angiogenic potential—have emerged as promising nanotherapeutics for chronic wound management, especially in diabetic settings (Cao et al., 2024).

#### 6.2.2 Corneal and uterine injuries

Adipose-derived stem cell Exos (ADSC-Exos) have demonstrated significant potential in promoting tissue repair and maintaining immune homeostasis due to their rich cargo of growth factors, microRNAs, and immunomodulatory molecules. Recent studies have highlighted the advantages of integrating ADSC-Exos with MNs delivery systems to enhance their therapeutic efficacy. For instance, Yu et al. reported the fabrication of a targeted MNs platform in which ADSC-Exos were modified with cornea-specific antibodies and subsequently delivered (Yu et al., 2024). This approach markedly accelerated corneal wound healing by enhancing epithelial regeneration and reducing inflammatory infiltration, offering a minimally invasive alternative to conventional treatments for ocular surface injuries.

In parallel, Wang et al. developed a Janus-structured MNs loaded with MSC-Exos for the treatment of intrauterine adhesions (Wang et al., 2024d). This localized delivery not only inhibited fibrotic adhesion formation but also promoted endometrial angiogenesis, epithelial proliferation, and restored hormonal responsiveness, thereby facilitating functional uterine regeneration.

#### 6.2.3 Spinal cord injurie

**Spinal cord injury** is a severe neurological disorder involving neuronal loss, axonal damage, and glial scar formation, often resulting in lasting functional deficits. While Exos-based therapies offer promise for neural repair by modulating inflammation and promoting regeneration, their clinical translation is hindered by challenges in achieving sustained and localized delivery to the lesion site.

To address this, Han et al. developed a HFMNs incorporating MSC-Exos derived from three-dimensional cultured MSCs (Han et al., 2022). When applied to injured spinal cord of rats, the HFMNs platform enabled continuous, localized release of MSC-Exos directly into the lesion area. This delivery system significantly attenuated the post-injury inflammatory response and inhibited excessive glial scar formation, which is a key barrier to axonal regeneration.

### 6.3 Ischemia-Reperfusion Injury

Ischemia-reperfusion injury (IRI) is a common pathological process in both cardiovascular and cerebrovascular diseases. Among them, myocardial infarction (MI) remains one of the leading causes of morbidity and mortality globally. Preventing post-infarction fibrosis and promoting functional recovery are critical therapeutic goals. In this context, Exos-MNs delivery systems have shown considerable promise.

**Fig. 6:**
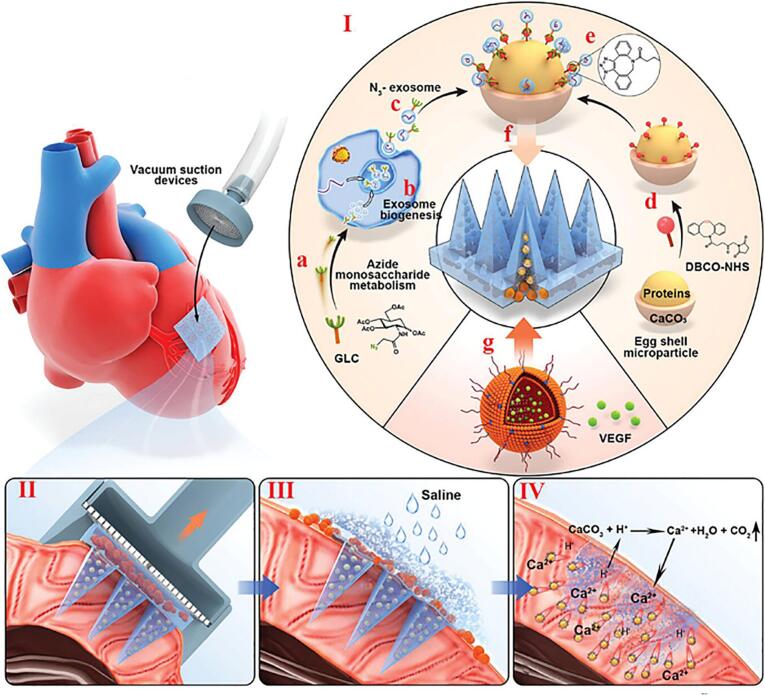
Schematic illustration of Exos-MNs for MI therapy. Reprinted with permission from (Wang et al., 2025). I. Design and fabrication of a dual-drug-loaded MNs featuring MSC-Exos and VEGF-loaded CaCO₃ microparticles. II–IV. Upon implantation and hydration in the infarcted myocardium, the MNs dissolve, release Exos, and initiate Ca^2+^-mediated VEGF release in response to the acidic environment, thereby enhancing angiogenesis and tissue repair.

In a mouse model of MI, Yuan et al. developed gelatin-based MNs loaded with human umbilical cord MSC-Exos (hUC-MSC-Exos) containing a miR-29b mimic—an anti-fibrotic microRNA (Yuan et al., 2023a, Yuan et al., 2023b). Localized MNs delivery significantly alleviated myocardial ischemia/reperfusion injury, attenuated inflammation, reduced infarct size, thickened the ventricular wall, and effectively inhibited fibrotic remodeling in the infarct border zone. Further advances were made by Wang et al., who designed a dual-compartment MNs for intelligent cardiac targeting (Fig. 6). The system utilizes a vacuum suction device for precise application to infarcted myocardium and is responsive to the acidic microenvironment typical of MI lesions. The MNs tips are functionalized with MSC-Exos for localized repair, while the patch base is embedded with CaCO₃/VEGF nanoparticles. Upon exposure to mild acidity, CaCO₃ decomposes to release Ca^2+^ and CO₂, facilitating patch adhesion and promoting angiogenesis through VEGF delivery, thereby enhancing cardiac tissue regeneration and functional recovery (Wang et al., 2025).

In addition to cardiac repair, Exos-MNs have demonstrated therapeutic potential in cerebral ischemia-reperfusion injury. Zhang et al. reported that MNs-assisted delivery of MSC-Exos significantly increased local Exos concentration in the ischemic brain region, promoted neuronal recovery, and minimized the systemic side effects commonly associated with intravenous Exos administration (Zhang et al., 2024). These findings support Exos-MNs as a promising strategy for integrated treatment of cardiovascular and cerebrovascular diseases.

### 6.4 Canker sores

Canker sores, or recurrent aphthous stomatitis, are superficial inflammatory lesions of the oral mucosa characterized by high prevalence, periodic recurrence, and uncertain etiology (Zeng et al., 2022). Owing to persistent mechanical stimulation from oral movements and continuous saliva secretion, conventional therapies—such as topical analgesics and anti-inflammatory agents—often fail to achieve effective and sustained drug delivery, thereby limiting therapeutic efficacy.

**Fig. 7:**
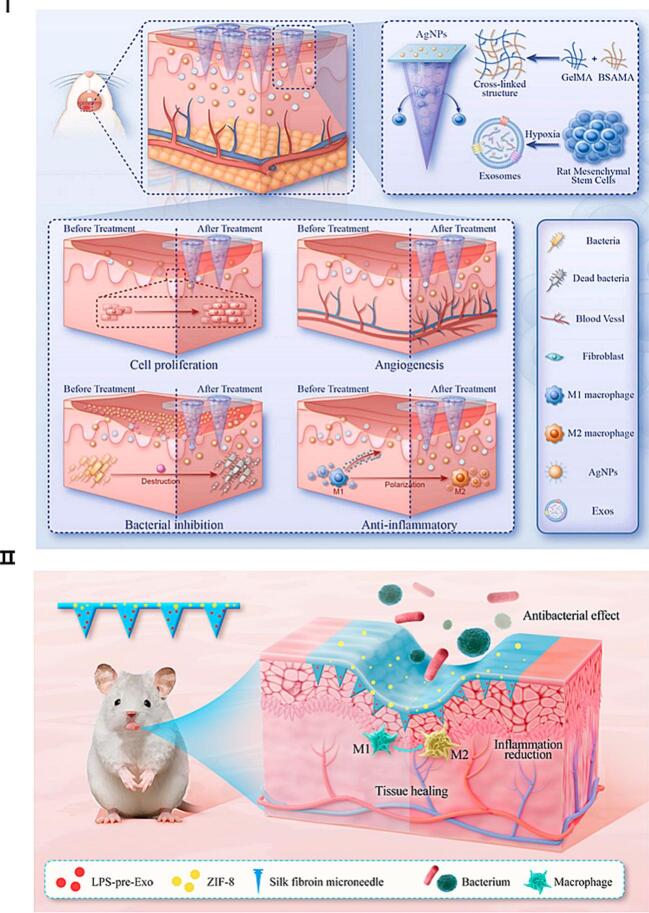
Exos-MNs for the treatment of canker sores. (I) Antibacterial composite protein MNs loaded with hypoxia-treated Exos for oral ulcer healing. Adapted from (Zeng et al., 2024). (II) Schematic diagram of silk fibroin MNs for treatment of oral ulcers (Ge et al., 2024). Adapted with permission from Ge et al. Copyright 2024, American Chemical Society.

To address these challenges, Zeng et al. developed a protein-based MNs system co-loaded with Exos and silver nanoparticles (AgNPs) (Fig. 7I)(Zeng et al., 2024). In this design, Exos are encapsulated in the needle tips for direct release into the lesion site, while AgNPs are distributed in the backing layer to exert antimicrobial effects. This dual-delivery strategy synergistically enhances antibacterial activity, suppresses inflammation, and promotes cellular proliferation and angiogenesis, collectively accelerating mucosal healing.

In a separate approach, Ge et al. introduced a silk fibroin MNs incorporating lipopolysaccharide (LPS)-preconditioned bone marrow MSC-derived Exos (LPS-pre-Exos) and ZIF-8 nanocarriers (Fig. 7 II)(Ge et al., 2024). The system exhibits excellent histocompatibility and achieves localized delivery of immunomodulatory Exos and antimicrobial agents, effectively reprogramming macrophages from the pro-inflammatory M1 phenotype to the reparative M2 phenotype. This immunoregulatory effect promotes tissue regeneration and provides sustained protection against bacterial infection in oral ulcer models.

### 6.5 Other

Exos-MNs have also been explored in the treatment of alopecia, offering a novel strategy for hair regeneration (Shi et al., 2022; Yang et al., 2019). Exos are rich in growth factors, extracellular vesicles, nucleic acids that can promote cell proliferation, differentiation, and tissue remodeling. When delivered via MNs, Exos can penetrate into the deep layers of the scalp, directly targeting hair follicle cells. This approach has been shown to activate follicular regeneration, enhance scalp microcirculation, and exert antioxidant, anti-inflammatory, and pro-angiogenic effects, thereby promoting hair growth in a multifaceted manner.

Additionally, Exos can serve as nanocarriers for therapeutic agents when combined with MNs, forming a dual delivery platform that enhances therapeutic precision. In this strategy, drugs are encapsulated within Exos, which are subsequently delivered transdermally via MNs. For instance, Exos loaded with TNF-α siRNA have been investigated for the treatment of rheumatoid arthritis, enabling targeted gene silencing within inflamed joints (Wen et al., 2024). Similarly, Exos encapsulating zycosyntropin have been applied in chronic pain management, achieving efficient delivery across the blood–brain barrier and into the central nervous system (Song et al., 2023). This design improves drug stability, enhances cellular uptake, and facilitates deeper tissue penetration. Table 3 summarizes representative Exos-MNs systems reported in recent years, highlighting differences in materials, Exos sources, therapeutic payloads, and disease treatments.

**Table 3:** Representative Exos-MNs delivery systems: an overview of MNs types, MNs materials, Exos sources, therapeutic payloads, and application areas.

MNs Type	MNs Materials	Exos sources	Therapeutic payloads	Application Area	Reference
HFMNs	GelMA/PEGDA	HUVECs	Tazarotene + Exos	Diabetic wound healing	(Yuan et al., 2022)
Double-layer DMNs	HAMA /PVA	M2 macrophages	Photosensitizer +PDA+ Exos	Diabetic wound healing	(Zeng et al., 2023)
DMNs	PVA	ADSCs	Anti-TNF-α-engineered Exos	Corneal injure	(Yu et al., 2024)
HFMNs	GelMA/HA /Acrylic acid/ Gelatin	MSCs	Exos	Intrauterine adhesion	(Wang et al., 2024d)
HFMNs	GelMA	3D-cultured MSCs	Exos	spinal cord repair	(Han et al., 2022)
DMNs	Gelatin	hUC-MSCs	Exos Containing miRNA-29b	Myocardial infarction	(Yuan et al., 2023b)
Implantable-tip MNs	PLGA	MSCs	VEGF-loaded nanoparticles + Exos	Myocardial infarction	(Wang et al., 2025)
HFMNs	Silk fibroin	LPS-preconditioned MSCs	ZIF-8 + LPS-pre-Exos	Canker sores	(Ge et al., 2024)
HFMNs	GelMA/BSAMA	Hypoxia-treated MSCs	AgNPs + Exos	Canker sores	(Zeng et al., 2024)
DMNs	HA/PVA	ADSCs	Chitosan lactate +Exos	Alopecia	(Shi et al., 2022)
DMNs	Keratin/HA	MSCs	UK5099+ Exos	Alopecia	(Yang et al., 2019)
DMNs	Trehalose	Milk	TNF-α siRNA-loaded Exos	Rheumatoid arthritis	(Wen et al., 2024a)
DMNs	GelMA	MSCs	Ziconotide-loaded Exos	Chronic pain	(Song et al., 2023)

## 7 Conclusion, challenges, and future prospect

Exos-MNs delivery represents a promising interdisciplinary strategy that bridges nanomedicine, regenerative biology, and transdermal therapeutics. By combining the minimally invasive administration and high skin penetration efficiency of MNs with the regenerative and immunomodulatory capabilities of Exos, Exos-MNs have demonstrated potential in various applications such as tissue repair, diabetic wound healing, MI treatment, and chronic pain management.

Despite these advancements, the clinical translation of Exos-MNs faces several critical challenges and unresolved knowledge gaps. First, the scalability of MNs fabrication compatible with the fragile nature of Exos remains underexplored. Many commonly used casting or molding techniques involve stressors—such as dehydration, UV crosslinking, or mechanical compression—that may compromise Exos membrane integrity and bioactivity. Moreover, the biodistribution, cargo release kinetics, and cellular uptake of Exos following transdermal delivery via MNs are still poorly understood, limiting the ability to rationally design systems for localized versus systemic delivery.

Additionally, batch-to-batch variability in Exos preparations—driven by differences in donor cells, culture conditions, and isolation techniques—poses a major hurdle for product standardization. The stability of engineered cargos, especially RNA and protein payloads, during MNs integration and long-term storage also requires careful optimization. From a regulatory perspective, Exos-MNs may be classified as combination products, involving both biologics and medical devices, which are subject to dual and complex approval pathways from agencies such as the FDA or EMA. This further complicates clinical translation.

To overcome these challenges, several strategic directions should be considered. First, the development of standardized Exos reference materials and release assays to ensure product consistency. Second, the use of synthetic Exos mimetics or hybrid vesicles to reduce heterogeneity while preserving biological function. Third, integrating diagnostic or sensing capabilities into MNs arrays, enabling real-time feedback on therapeutic release and tissue responses.

In summary, while Exos-MNs represent a highly promising platform for next-generation biologics delivery, their future success depends on coordinated progress in bioengineering, regulatory science, and translational research. With continued innovation and standardization, these hybrid systems may ultimately revolutionize both localized and systemic drug delivery in clinical settings.

## Funding

This work was supported by the Suzhou Municipal Health Commission (SZWJ2024a022).

## CRediT authorship contribution statement

**Lijie Zheng:** Writing – original draft, Conceptualization. **Jiting Sun:** Formal analysis. **Lusheng Wang:** Investigation. **Zhixian Ding:** Investigation. **Yu Tang:** Visualization. **Mike Dai:** Visualization, Conceptualization. **Heng Tang:** Writing – review & editing, Conceptualization.

## Declaration of competing interest

The authors declare that they have no known competing financial interests or personal relationships that could have appeared to influence the work reported in this paper.

## Data Availability

No data was used for the research described in the article.
